# Pollen transport networks reveal highly diverse and temporally stable plant–pollinator interactions in an Appalachian floral community

**DOI:** 10.1093/aobpla/plab062

**Published:** 2021-09-19

**Authors:** Daniel A Barker, Gerardo Arceo-Gomez

**Affiliations:** Department of Biological Sciences, East Tennessee State University, Johnson City, TN 37614, USA

**Keywords:** Community structure, interaction diversity, plant, pollinator networks, pollen, pollination success

## Abstract

Floral visitation alone has been typically used to characterize plant–pollinator interaction networks even though it ignores differences in the quality of floral visits (e.g. transport of pollen) and thus may overestimate the number and functional importance of pollinating interactions. However, how network structural properties differ between floral visitation and pollen transport networks is not well understood. Furthermore, the strength and frequency of plant–pollinator interactions may vary across fine temporal scales (within a single season) further limiting our predictive understanding of the drivers and consequences of plant–pollinator network structure. Thus, evaluating the structure of pollen transport networks and how they change within a flowering season may help increase our predictive understanding of the ecological consequences of plant–pollinator network structure. Here we compare plant–pollinator network structure using floral visitation and pollen transport data and evaluate within-season variation in pollen transport network structure in a diverse plant–pollinator community. Our results show that pollen transport networks provide a more accurate representation of the diversity of plant–pollinator interactions in a community but that floral visitation and pollen transport networks do not differ in overall network structure. Pollen transport network structure was relatively stable throughout the flowering season despite changes in plant and pollinator species composition. Overall, our study highlights the need to improve our understanding of the drivers of plant–pollinator network structure in order to more fully understand the process that govern the assembly of these interactions in nature.

## Teaser

Floral visitation has been used to characterize plant–pollinator interactions even though it ignores differences in the quality of floral visits. Insect pollen loads on the other hand can provide more accurate description of the functional consequences of these interactions but this has been little studied. The strength and frequency of plant–pollinator interactions may also vary within a single season further limiting our understanding of the consequences of plant–pollinator interactions. We compared plant–pollinator structure using floral visitation and pollen transport data and evaluate within-season variation in community structure in a diverse plant–pollinator community. Pollen transport data revealed a larger diversity and up to two times as many plant–pollinator interactions. Pollen transport network structure was stable throughout the flowering season despite changes in plant and pollinator species composition. Our study highlights the need to improve our understanding of community-level plant–pollinator interactions and how they are structured in nature.

## Introduction

Plant–pollinator interactions typically take place within large and diverse communities where plant and pollinator species can interact directly and/or indirectly (Roughgarden 1979; Thompson 1999; Jordán *et al.* 2008; Burkle and Alarcón 2011; Carstensen *et al.* 2014). The complex make-up of these interactions can in turn affect the stability of natural communities (Bascompte *et al.* 2003; Olesen *et al.* 2007; Stouffer and Bascompte 2011; Valido *et al.* 2019). Knowledge of the overall structure, as well as the drivers and consequences of community-wide plant–pollinator interactions is thus essential for gaining a complete understanding of the processes that shape natural communities and how these will respond in the face of increasing human disturbances (Memmott *et al.* 2004; Katariya *et al.* 2017; Pérez-Méndez *et al.* 2018).

Network theory has been successfully used to describe complex community-level plant–pollinator interactions (Memmott 1999; Bascompte *et al.* 2003; Memmott *et al.* 2004; Palla *et al.* 2005; Mason and Verwoerd 2006; Popic *et al.* 2013; Pocock *et al.* 2016) and has revealed key generalities in the assembly of plant–pollinator communities (Ollerton *et al.* 2006; Jędrzejewska-Szmek and Zych 2013; Pocock *et al.* 2016). These studies have uncovered plant–pollinator communities that are highly connected, generalized, nested and robust to human disturbances (e.g. Dupont *et al.* 2003; Fortuna and Bascompte 2006; Burgos *et al.* 2007; Piazzon *et al.* 2011; Nielsen and Totland 2014). However, recent studies have suggested that plant–pollinator networks, and our interpretation of their structural patterns, are limited in that they typically overlook the temporal dynamics of these interactions (CaraDonna and Waser 2020; Thomson 2021). It is also not well known how patterns of pollinator visitation reflect other key aspects of the pollination process such as pollen transport and deposition on stigmas (Parra-Tabla and Arceo-Gómez 2021; Thomson 2021). These two shortcomings thus hamper our ability to fully evaluate the role of plant–pollinator interactions in the ‘stability’ of natural communities, hence preventing advancing towards a more predictive understanding of the causes and functional consequences of plant–pollinator network structure in nature.

Specifically, plant–pollinator interactions have been typically characterized based on the observation of floral visitation (hereafter FV) to help establish links between plants and pollinators in a network context (e.g. Alarcón *et al.* 2008; Baldock *et al.* 2011; Vazquez *et al.* 2012; Lázaro *et al.* 2008). However, utilizing FV alone likely ignores differences in the quality of floral visits (i.e. transport and deposition of pollen), and may overestimate the number and functional importance of ‘pollination interactions’ (from a plant fitness perspective; e.g. King *et al.* 2013; Parra-Tabla and Arceo-Gómez 2021; Thomson 2021). For instance, a recent study showed that when pollinator efficiency (contribution to pollen transport) is considered, only half of pollinators and 27 % of all interactions in a network contribute to plant fitness (de Santiago-Hernández *et al.* 2019). It is also possible that rare (i.e. infrequent and difficult to observe), but functionally important (e.g. high pollen transport) interactions are overlooked when relying on FV alone (Jędrzejewska-Szmek and Zych 2013). These limitations of using FV may in turn obscure our understanding of the overall structure and functional consequences of plant–pollinator networks. For instance, it was recently suggested that an increase in plant–pollinator specialization (based on FV) within nested communities could lead to higher levels of heterospecific pollen transfer (Arceo-Gomez *et al.* 2020), hence decreasing pollination quality (Morales and Traveset 2008). This because in nested plant–pollinator networks specialized plant species tend to interact more frequently with generalist pollinators that in turn visit many other plant species (Arceo-Gomez *et al.* 2020). Thus, networks derived from the use of FV alone may lead to an incomplete characterization of the fitness consequences of plant–pollinator network structure and consequently of its implications for community robustness and resilience (Alarcón 2010; Jędrzejewska-Szmek and Zych 2013; Popic *et al.* 2013).

Using information on the size and identity of pollen loads carried by floral visitors (i.e. pollen transport) to infer plant–pollinator network structure may help overcome the limitations of the use of FV (Alarcón 2010; Tur *et al.* 2014). For instance, pollen transport (hereafter PT) data may allow the identification of floral visitors that contribute very little to the pollination process (i.e. do not carry pollen) and thus may be functionally irrelevant from a plant fitness perspective (de Santiago-Hernández *et al.* 2019). Furthermore, PT data may increase the likelihood of detecting rare plant–pollinator interactions that are critical for plant fitness, but that are difficult to observe in the field (Jędrzejewska-Szmek and Zych 2013). In the few studies conducted to date (e.g. Alarcón 2010; Jędrzejewska-Szmek and Zych 2013) PT networks have been shown to capture a larger diversity and number of interactions compared to FV networks and thus may have the potential to provide a more accurate depiction of the functional relevance of plant–pollinator interactions in a community (Bosch *et al.* 2009; Alarcón 2010; Jacobs *et al.* 2010; Olesen *et al.* 2011a). Thereby revealing patterns of network structure that may be more informative for assessing overall community robustness and stability. However, how plant–pollinator network structural properties based on FV differ from those constructed from PT remains little studied (but see Alarcón 2010; Jędrzejewska-Szmek and Zych 2013).

A second key limitation in most plant–pollinator network studies has been the pooling of interactions across one or multiple flowering seasons (e.g. Basilio *et al.* 2006; Olesen *et al.* 2008, 2011b), thus assuming these interactions are static over time (Thomson 2021). A few studies however have shown that plant–pollinator networks can vary substantially between years with as much as 25 % species turnover in plant and pollinator species composition (e.g. Petanidou *et al.* 2008). Furthermore, temporal turnover in species interactions may not only occur between different years but may also occur within the same flowering season (e.g. CaraDonna *et al.* 2017; Thomson 2021), although this has been far less explored. Within-season variability in network structure could occur due to variation in species flowering phenology (flowering time) as well as due to variation in the timing of activity of pollinators during the flowering season (Olesen *et al.* 2008; CaraDonna *et al.* 2017). In this case, plant–pollinator interactions can be expected to change as pollinator preferences change with changes in the availability of floral resources and/or as new pollinator species emerge (Fowler *et al.* 2016; CaraDonna *et al.* 2017; Thomson 2021). Understanding how plant–pollinator network structure changes at finer temporal scales such as within a single season and how patterns of FV reflect those of PT will greatly help advance our understanding of the drivers and functional consequences of changes in plant–floral visitor network structure.

In this study we evaluate differences in plant–floral visitor network structure (e.g. nestedness, modularity, connectance) based on FV and PT data and evaluate how the structure of PT networks changes a different time points within a single season in a plant–pollinator community in southern Appalachia. We ask the following specific questions: (i) do network structure derived from PT data differs significantly from FV networks? and (ii) does PT network structure differ between the early, middle and late portions of the flowering season?

## Materials and Methods

### Study site

The study was conducted in Hampton Creek Cove State Natural Area in Tennessee, USA (36°08.843′N, 82°02.794′W, elevation: 971 m). The site is a 1.87-ha field undergoing secondary succession with a mix of animal-pollinated annual and perennial, as well as native and non-native plants inhabiting the site **[see****Supporting Information—Fig. S1****]**. The study site is bordered by a mixed deciduous forest. Flowering starts in late April and continues into early September (Daniels and Arceo-Gómez 2020). Temperature at the study site ranges from 22 to 34 °C during the day. There are several federally recognized endangered and threatened plant species located at the study site including: Blue Ridge Goldenrod (*Soldiago spithamaea*), Roan Mountain Bluet (*Houstonia montana*) and Spreading Avens (*Geum radiatum*) and thus this plant–pollinator community is also of potential conservation concern.

### Pollinator collection

To sample the pollinator community four 1 × 40 m transects were set up at the study site. Pollinators were sampled by walking each transect at a constant pace collecting all insects observed visiting flowers until a maximum of 60 (range 45–60) floral visitors were sampled each day. Since in plant–pollinator networks the unit of ‘sampling’ are the individual interactions we established a maximum number of floral visitors (i.e. 60) collected each day in order to standardize for sampling effort (i.e. number of interactions observed) and thus be able to evaluate differences in network structure among different time periods (early, middle, late flowering season). This standardization further avoids large differences in network size which have been shown to greatly influence network structure and thus allowed us to safely compare between distinct time periods without biasing our results. Sampling took place between May and August 2019 during peak pollinator activity between 8:00 AM and 3:00 PM (Daniels and Arceo-Gómez 2020) for 21 days across 13 weeks. We sampled 7 days per time period, i.e. early season (ES), middle of the season (MS) and late in the season (LS). All insects were collected with butterfly nets when they were observed making contact with the floral reproductive structures (anthers and stigma). Upon collection, insects were stored in individual containers and placed in a cooler until processing in the laboratory. The identity of the plant species where each pollinator was captured was recorded.

A total of 917 insects were collected and identified to the lowest taxonomic group possible using several insect identification guides (Field Guide to Insects of North America, Peterson Field Guides Insects, Field Guide to Insects and Spiders of North America). Where species identity could not be confirmed, individuals were assigned to morphogroups or recognizable taxonomic units (Vanbergen *et al.* 2014; Oliver and Beattie 2016). In total 103 insect morphogroups were found at the study site **[see****Supporting Information—Fig. S2****]**.

### Pollen load sampling

Insect pollen loads were sampled by swabbing the body of each floral visitor collected with fuchsin jelly cubes (Beattie 1971; Kearns and Inouye 1993). The fuchsin jelly was made by mixing 175 mL of distilled water to 150 mL of glycerol and 50 g of gelatin which was then mixed with basic fuchsin crystals (Beattie 1971). The jelly was cut into approximately 3 × 3 × 1 mm cubes and then applied to the top and bottom of the thorax and abdomen, the head and mouth parts, antennae if present, and to the legs of each insect. The corbiculae of bee species was avoided as the pollen located within it is not typically available for pollination (i.e. deposition on the stigma; Horskins and Turner 1999; Popic *et al.* 2013; Johnson and Ashman 2019). Each appendage was swabbed three times to standardize sampling. Fuchsin jelly swabs with pollen samples were then placed on microscope slides and melted over a hot plate before being sealed under a glass coverslip. The pollen loads of all 917 insects were sampled.

After pollen samples had been mounted, each sample was observed under a microscope and all pollen in the sample was identified and counted. Identification of pollen grains was done with the aid of a pollen reference library constructed for all plant species at the study site (also see Daniels and Arceo-Gómez 2020). Quantification and identification of pollen grains was done with a compound light microscope at 400× magnification. If identification of pollen could not be confirmed to match any of the species present at the studied community, they were recorded as unknown and were not included in the network (only 4 % of total pollen counted). In the few instances where two plant species had similar pollen morphology as determined by the pollen reference library, these were combined into one group (four species were dived into two pollen groups). In total, 214 346 pollen grains were counted and identified to 48 species of plants **[see****Supporting Information—Fig. S1****]**.

### FV and PT networks

Plant–floral visitor interactions were characterized using PT and FV data collected across the entire flowering season (13 weeks). Within each interaction matrix (PT and FV), the frequency of plant–floral visitor interactions was determined by the number of observed flower visits (FV) and the average pollen load (PT) found on the body of each floral visitor species. The number of flower visits was determined from the number of insect collections on flowers of a given plant species. For the PT network, the average number of pollen grains of each plant species found on each insect morphogroup was used in place of the number of floral visits. However, it has been suggested that pollinators can pick up pollen grains during ‘accidental’ visits to flowers (e.g. flowers that pollinators do not typically visit), or pick up pollen grains from more than one species on a single flower, thus overestimating the ‘functionally relevant’ number of interactions (Ne’Eman *et al.* 2010). To account for ‘incidental’ PT, we are considering interactions with an average of five or more pollen grains of a specific plant species as actual pollination events (less than five grains has been considered incidental pollination; e.g. Johnson and Ashman 2019). We further improved the reliability of our estimate by only removing interactions of ≤5 pollen grains that also constituted less than 5 % of the total pollen load carried by each insect species. By doing this we avoided removing interactions that may be ‘functionally relevant’ but where pollen grains are naturally transported in small amounts (e.g. pollinator size constraints). For instance, five pollen grains could be 50 % of total pollen load if pollinators only carry 10 total pollen grains and thus this interaction would not be considered ‘incidental’. In total, 336 ‘incidental’ plant and pollinator species interactions were removed from the PT network. If no interaction (visit/pollen) was observed between a plant and insect species a zero was recorded.

### Temporal variation in PT networks

Within-season variability was only assessed using PT data because these have been shown here (see Results) and in other studies (e.g. Alarcón 2010; King *et al.* 2013; de Manincor *et al.* 2020) to provide the most complete, diverse and reliable characterization of functionally relevant plant–pollinator interactions in a community. Thus, from a plant fitness perspective, changes in PT network structure across time may be more ecologically relevant than changes in flower visitation networks. We evaluated PT network structure across early (ES), middle (MS) and late flowering season (LS) by constructing plant–floral visitor networks for each individual week (13 total weeks) and then categorizing each week as early, middle or late flowering. Each time period was then composed of 4–5 replicates per week (i.e. ES weeks 1–4, MS weeks 5–8 and LS weeks 9–13). We divided the growing season into 4- to 5-week time periods (ES, MS, LS) as they coincide with ample plant species turnover in the studied community (D. A. Barker, pers. obs.; **see****Supporting Information—Fig. S3**). Previous studies have shown that differences in plant–floral visitor network structure can be expected as a result of high plant and pollinator species turnover (CaraDonna *et al.* 2017; CaraDonna and Waser 2020). For instance, at our study site, *Jacobaea vulgaris* (ES), *Glechoma hederacea* (MS) and *Achillea millefolium* (LS) were observed flowering in only one of the three time periods studied (Daniels and Arceo-Gomez 2020; **see****Supporting Information—Fig. S3**).

### Statistical analysis

We evaluated overall differences between FV and PT network structure in two ways. First, we performed mixed models using the 13 weekly networks (see above) as replicates of FV and PT networks (26 total networks). Although networks are built from samples collected at the same study site, weekly networks represent a distinct plant and pollinator community as the abundance and diversity of plant and pollinator species changes week to week (**see****Supporting Information—Fig. S3**; also see CaraDonna and Waser 2020). This not only results in weekly changes in plant and pollinator species composition but can also lead to a significant turnover of interactions (Carstensen *et al.* 2016; CaraDonna *et al.* 2017). Weekly networks thus capture distinct plant–pollinator communities and the interactions among a specific subset of plant and pollinator species. Since we are interested in sampling biological communities and not physical locations weekly plant and pollinator communities may be considered distinct replicates of FV and PT networks. We evaluated differences in overall network metrics using network type (FV or PT) as a fixed effect and sampling week as a random effect. We evaluated differences in network structure by estimating weighted network connectance, average links per species, weighted nestedness, specialization (H2) and modularity. Specifically, connectance represents the proportion of realized interactions out of the total number of possible interactions, while the number of links per species represents the average number of interactions for each species in the network, both reflect differences in the size and complexity of the network (Bascompte *et al.* 2003; Blüthgen *et al.* 2006; Olesen *et al.* 2007). Nestedness reflects the degree to which specialist species interact with subsets of the species interacting with generalists, while modularity reflects the existence of highly connected, non-overlapping groups of species (i.e. plant–pollinator interactions that occur within certain groups/modules and no others; Bascompte *et al.* 2003). Network specialization (H2) on the other hand reflects differences in the degree of niche partitioning across species (i.e. specialization) between networks (Dunne *et al.* 2002; Blüthgen *et al.* 2006; Olesen *et al.* 2007). All these metrics were generated using the bipartite package in R and have been commonly used to describe plant–pollinator network structure (e.g. Dunne *et al.* 2002; Basilio *et al.* 2006; Petanidou *et al.* 2008; Bosch *et al.* 2009; Alarcón 2010; Olesen *et al.* 2011b). Furthermore, these network properties are dependent on the number, identity and frequency of the interactions among network participants, which have been shown to vary temporally (Ponisio *et al.* 2017; Valido *et al.* 2019; CaraDonna and Waser 2020).

Second, we evaluated differences between FV and PT networks using Procrustes analysis (Alarcón 2010; Johnson and Ashman 2019). For this, we used two networks constructed from pooled data collected across all 13 weeks (i.e. one PT and one FV network). Procrustes analysis evaluates differences in network shape using corresponding landmarks (i.e. network nodes/species) within the networks. (Wang *et al.* 2010; Demayo *et al.* 2011; Piazzon *et al.* 2011; Dehling *et al.* 2016). The congruence in the position of the nodes (i.e. species) between two networks is then reduced by rotating, inverting, enlarging or reducing the networks (Alarcón *et al.* 2008). As such, Procrustes analysis evaluates differences in network structure taking into account the identity and position of each species (landmarks) within the networks (Alarcon *et al.* 2008; Wang *et al.* 2010; Demayo *et al.* 2011; Piazzon *et al.* 2011; Dehling *et al.* 2016). This approach differs from our previous analyses in that mixed models only evaluate changes in overall network structure irrespective of species identities/roles within the network. On the other hand, significant differences using Procrustes analyses reflect network structural differences that result from changes in individual species position (role) within a network.

We also evaluated if observed network metrics in PT and FV networks, constructed from data across all 13 weeks (i.e. one PT and one FV network), are the result of a random assembly using null model analysis. For this, we used the ‘vaznul’ algorithm in the bipartite package, as it is least sensitive to the abundances of interactions (Vázquez *et al.* 2007; Dormann *et al.* 2009; Souza *et al.* 2018). We generated 9999 random networks maintaining the number of interactions constant and compared the observed network metrics with those of the randomly generated networks using Spearman’s correlation coefficient to generate *z*-scores for each metric (Vázquez *et al.* 2007).

To evaluate within-season variation in PT network structure (using the same network metrics described above) we performed a mixed model using the 13 weekly networks (see above) as replicates of early (4 weeks), middle (4 weeks), late (5 weeks) flowering season. Flowering period (ES, MS, LS) was considered as a fixed effect and individual week as random effect. As above, we further conducted Procustes analyses to evaluate within-season (ES, MS, LS; one network per time period) differences in PT network structure considering changes in species position within the network. Interaction sampling completeness (including all interactions observed) was evaluated via rarefaction analyses for overall FV and PT networks and for each sampling period (ES, MS, LS) using EstimateS (ver. 9.1.0). For this, we used the Jackknife and bootstrap richness estimation methods as these have been shown to perform best at small (21 days) but intense sampling efforts (Poulin 1998; Walther and Morand 1998), thus avoiding underestimating sampling completeness compared to other estimators (Macgregor *et al.* 2017). We compared both richness estimators (Jackknife and bootstrap) with the observed number of unique plant–pollinator species interactions observed. All bipartite networks and network metrics were generated using the bipartite package in R (Dormann *et al.* 2008; R Core Team 2017). Residuals were normally distributed in all models (Shapiro–Wilkes, *P* > 0.05).

## Results

Rarefaction analysis showed that our sampling captured between 62 and 79 % (Jackknife and bootstrap, respectively) of all FV and between 64 and 81 % of all PT interactions in the community **[see****Supporting Information—Fig. S4****]**. Of the 917 insects collected and identified, Hymenoptera and Diptera accounted for 44 and 26 %, respectively. Hemiptera, Coleoptera, Lepidoptera and Orthoptera represented 8.5, 14.7, 6 and 0.2 %, respectively. Although Hemipterans are not typically considered pollinators, our data showed that these may transport pollen between flowers as their pollen loads exceeded 100 grains per individual on average. Hence, these were included in the networks.

### Congruence between FV and PT networks

**Figure 1.**
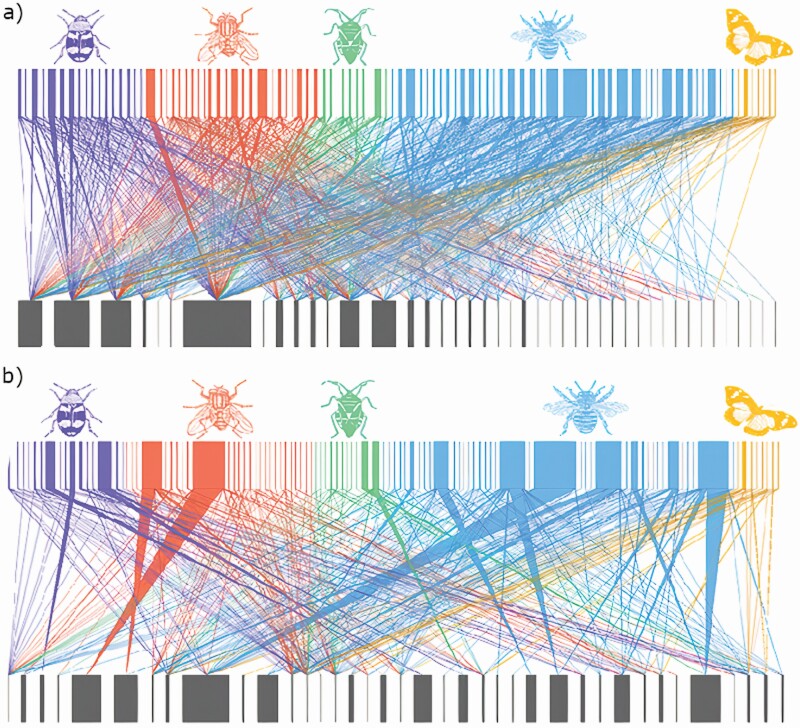
Plant–pollinator interaction networks constructed based on (A) PT and (B) FV data. Networks were constructed from pooled data across all 13 weeks. Insect morphogroups are represented by nodes on the top (purple = Coleoptera, red = Diptera, green = Hemiptera, blue = Hymenoptera, orange = Lepidoptera) and plants at the bottom. Interactions are represented by the lines between nodes. The thickness of the lines reflects the frequency of those interactions.

Pollen transport and FV networks contained 554 and 357 unique interactions, respectively (Fig. 1). There were 95 (PT) and 103 (FV) floral visitors interacting with 43 (PT) and 39 (FV) plant species (Fig. 1). Pollen transport and FV network structure differed significantly in weighted connectance (*F*_24, 1_ = 5.73, *P* = 0.02), average links per species (*F*_24,1_=65.84, *P*< 0.01), and weighted nestedness (*F*_24, 1_ = 9.16, *P* = 0.006) (Table 1). There was also a marginal difference in specialization (H2) (*F*_24, 1_ = 3.76, *P* = 0.06) but no differences in network modularity (*F*_24, 1_ = 0.04, *P* = 0.9; Table 1). Null model analyses showed that all network properties were significantly different than what would be expected by random (*P* < 0.05). Procrustes analysis showed no significant differences between PT and FV networks (*PSS* = 0.96, *P* = 0.2).

**Table 1.** Average (± SD) and range (min–max) for each network structural metric (links per species, weighted nestedness, weighted connectance, modularity and specialization [H2]) generated for FV and PT networks and for early (ES), middle (MS) and late (LS) flowering periods within a single season. The number of replicates (weekly networks) for each network type is provided in parentheses. Significantly different values (*P* < 0.05) are shown in bold, *P = 0.06.

		FV (13)	PT (13)	ES (4)	MS (4)	LS (5)
Links per species	Mean	**1.11**	**2.01**	2.02	2.03	1.98
	SD	0.14	0.37	0.29	0.32	0.53
	Min/max	0.89/1.31	1.2/2.5	1.71/2.31	1.82/2.5	1.2/2.5
Weighted nestedness	Mean	**0.3**	**0.45**	0.46	0.4	0.5
	SD	0.13	0.12	0.06	0.19	0.1
	Min/max	0.08/0.50	0.23/0.58	0.40/0.52	0.23/0.57	0.34/0.58
Weighted connectance	Mean	**0.07**	**0.84**	0.08	0.09	0.08
	SD	0.03	0.01	0.01	0.01	0.02
	Min/max	0.02/0.10	0.06/0.10	0.06/0.09	0.08/0.1	0.06/0.1
Modularity	Mean	0.52	0.52	0.5	0.54	0.53
	SD	0.12	0.09	0.13	0.05	0.09
	Min/max	0.34/0.70	0.35/0.69	0.35/0.62	0.47/0.60	0.46/0.69
Specialization (H2)	Mean	0.77*	0.63*	0.65	0.67	0.59
	SD	0.23	0.1	0.05	0.08	0.14
	Min/max	0.37/0.99	0.35/0.69	0.59/0.72	0.54/0.71	0.48/0.82

### Within-season variation

**Figure 2.**
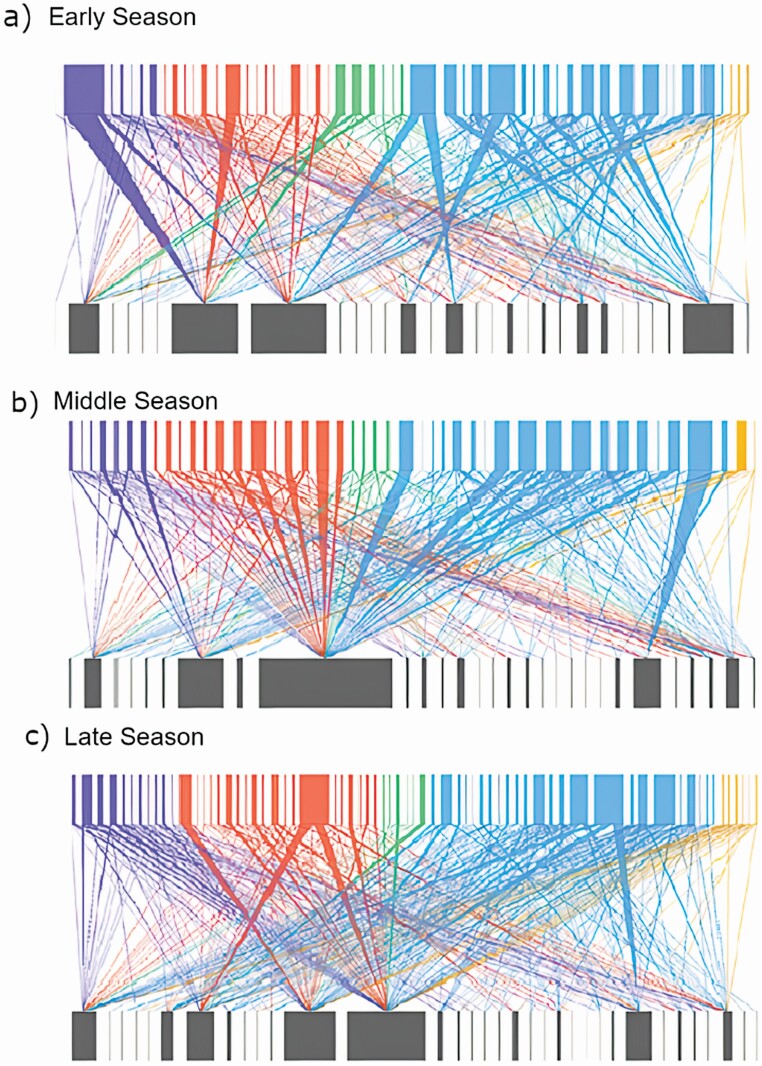
Plant–pollinator interaction networks constructed from PT data collected (A) early in the season (ES): mid-May to early June, (B) middle of the season (MS): June to early July and (C) late season (LS): July to mid-August. Each time period (network) consists of pooled data across 4-/5-week intervals. Insect morphogroups are represented by nodes on the top (purple = Coleoptera, red = Diptera, green = Hemiptera, blue = Hymenoptera, orange = Lepidoptera) and plants on the bottom. Interactions are represented by the lines between nodes. The thickness of the lines reflects the frequency of those interactions.

Rarefaction analysis showed that our sampling captured between 63 and 80 % (Jackknife and bootstrap respectively) of all expected plant–pollinator interactions across in each of the three time periods, i.e. early, middle and late in the flowering season **[see****Supporting Information—Fig. S4****]**. Early (ES), middle (MS) and late season (LS) networks contained 260, 229 and 333 unique interactions, 51, 45 and 68 floral visitors and 29, 29 and 34 plant species, respectively (Fig. 2). There was no within-season differences in the average number of links per species (*F*_10, 2_ = 0.02, *P* = 0.98), weighted nestedness (*F*_10, 2_ = 0.65, *P* = 0.5), weighted connectance (*F*_10, 2_ = 1.39, *P* = 0.29), specialization (H2; *F*_10, 2_ = 0.61, *P* = 0.56) and modularity (*F*_10, 2_ = 0.21, *P* = 0.816; Table 1). Procrustes analysis, however, indicated that the ES, MS and LS networks were all significantly different from each other (*PSS* > 0.91, *P <* 0.05 for all).

## Discussion

Our results show that PT networks may provide a more complete depiction of the diversity and complexity of plant–floral visitor interactions in a community. Specifically, PT networks captured 38 % more unique interactions and twice as many links per species compared to the FV network (4.6 vs. 2.7; Table 1). Moreover, six insect species ‘dropped out’ of the plant–floral visitor network when pollen loads were considered, likely because these species do not contribute to PT and therefore may be functionally irrelevant in a pollination context. Surprisingly, this was the case for the fiery skipper *Hylephila phyleus* (Lepidoptera) who was never found carrying pollen even though it was somewhat abundant at the site (i.e. multiple individuals were collected and swabbed). It is important to point out, however, that interactions that we considered here as ‘functionally irrelevant’ from a pollination perspective can be biologically relevant in other contexts. For instance, some of these can be antagonistic interactions (e.g. nectar robbers; Thomson 2021), may contribute to viral/microbial transport or may contribute to pollinator food provisioning, all of which can directly or indirectly impact plant or pollinator fitness (Bronstein 2001; Morris *et al.* 2019). In our study, for example, we observe six insects that do not seem to contribute to pollination (i.e. insects observed visiting flowers but not carrying pollen) but may be serving other important ecological functions in the community **[see****Supporting Information—Fig. S5****]**. In a similar manner, four plant species were included in the PT network but were not present in the FV network. This likely occurred because visits to these plant species are infrequent and therefore hard to observe. For example, *Prunella vulgaris* and *Solanum carolinense* were not observed receiving any insect visits, although pollen from both plants was found on the bodies of 20 and 2 pollinator morphogroups, respectively. Overall, our results suggest that PT networks are more accurate descriptors of the diversity and frequency of community-level plant–pollinator interactions.

Our results further show that FV and PT networks differ in their overall structure, although these differences seem to be only related to the strength of network structural properties. For instance, while both network types were significantly nested (compared to random assembly), the PT networks had significantly higher nestedness values compared to the FV networks (Table 1). This difference may be due to an increase in number of interactions in PT networks compared to the FV network (Dupont *et al.* 2003). Interestingly, FV networks were not significantly different from PT networks when evaluated via Procrustes analysis, suggesting that species’ position/roles within the networks do not change between the two network types. These results combined suggest that event though PT networks capture a much larger number and diversity of interactions, FV networks may still be reliably describing the overall assembly of plant–pollinator interactions in terms of their overall structure. In a recent study, de Manincor *et al.* (2020) showed similar results and concluded that FV networks provide a suitable representation of plant–pollinator interactions despite the fact that they capture significantly less interactions compared to PT networks. In our study, however, we did observed differences in the overall strength of structural network properties with potential implications for network structure and stability. For instance, increasing network nestedness and connectance have been associated with increasing community stability (Memmott and Waser 2002; Bastolla *et al.* 2009; Thébault and Fontaine 2010; Nielsen and Totland 2014) and tolerance to human-mediated disturbances (Dunne *et al.* 2002). Higher levels of nestedness have also been associated with increased resistance to species loss (Bascompte *et al.* 2003; Burgos *et al.* 2007). Thus, a strong reliance on FV networks may still limit our predictive understanding of the effect of human disturbances on plant and pollinator communities.

Our results also show that plant–pollinator networks based on PT although much more diverse, their structure is relatively stable throughout the flowering season. None of the network metrics we evaluated differed significantly between early, middle and late flowering season. This stability in overall network structure is intriguing given that the diversity and identity of the interacting plant and pollinator species (and their interactions) changed considerably along the season at the study site. For example, *J. vulgaris* was the dominant plant species early in the season (mid-May) but it is completely replaced in early June by *Crepis capillarus*, and this species is in turn replaced by *A. millefolium,* at the end of the season **[see****Supporting Information—Fig. S3****]**. In fact, Procustes analysis revealed changes in individual species roles within the network throughout the flowering season likely reflecting the high plant species turnover observed over time. However, despite this high plant species turnover, overall PT network structure remained constant. For instance, average number of links per plant species (1.98–2.03), nestedness (0.4–0.5) and modularity (0.5–0.53) remained relatively stable throughout the season (Table 1). These results may hence suggest the existence of an overarching ‘blueprint’ in the structure of plant–pollinator interactions regardless of community species composition (also see Alarcón *et al.* 2008). A similar trend has also been observed at larger time scales, where network structure remains constant across years despite a vast turnover in the identity of the interacting species (Alarcón *et al.* 2008). These results, however, differ from recent work by CaraDonna and Waser (2020) which showed changes in network structure with plant and pollinator species turnover. The apparent constancy in plant–pollinator network structure, like the one observed here, however, has been attributed to the dominance of generalist species that serve as ‘anchor’ or ‘bridge’ species that help maintain network structure (Olesen *et al.* 2011b; Koski *et al.* 2015). In our study, *Trifolium pratense* and *Trifolium repens* were both abundantly available throughout the flowering season and could help serve as ‘bridge’ maintaining network structure despite further changes in plant species composition (e.g. Koski *et al.* 2015). However, to our knowledge, few studies have evaluated structural changes in plant–pollinator network structure at short temporal scales (within a single season; e.g. Saavedra *et al.* 2016; CaraDonna and Waser 2020). Thus, we highlight the need for more studies that evaluate the causes and consequences of fine-scale temporal changes (e.g. within a season and within a day) in plant–pollinator network structure in order to more fully understand the process that governs the assembly of these interactions in nature. Overall, our results emphasize the need for studies that evaluate the drivers of plant–pollinator network stability beyond FV and across changing plant and pollinator communities in space and time. Such studies would help improve our predicative understanding of the consequences of human-mediated disturbances on natural plant and pollinator communities.

## Supporting Information

The following additional information is available in the online version of this article—

Figure S1. Plant species documented at the study site. 

Figure S2. Insect species documented visiting flowers at the study site. 

Figure S3. Phenologies of plant species at the study site throughout the sampling period (13 weeks). 

Figure S4. Rarefaction curves representing sampling completeness for each network type a) floral visitation-whole, b) pollen transport-whole and for each time period within a season, c) early season (ES), d) middle season (MS) and e) late season (LS). 

Figure S5. Bipartite network of ‘non-pollinating’ interactions between plants and insects. The network was generated by subtracting ‘pollen-transport’ interactions from the ‘floral visitation’ network. 

plab062_suppl_Supplementary_Materials

## Data Availability

All data used for analysis in this publication can be found in the following Dryad repository (doi:10.5061/dryad.f1vhhmgxk).

## Notes

**Populations & Communities. Chief Editor**: Jean Burns
