# Supplementary material for: Pollen transport networks reveal highly diverse and temporally stable plant–pollinator interactions in an Appalachian floral community
Source: AoB Plants. 2021 Sep 19;13(5):plab062. doi: 10.1093/aobpla/plab062 (PMC8508780; doi:10.1093/aobpla/plab062)

Figure S1- Plant species documented at the study site. Table lists the Family, Genus, and Species of flowering plants at Hampton Creek Cove State Natural Area.

**Family**	**Genus**	**Species**
Adoxaceae	*Sambuca*	*canadensis*
Apocynaceae	*Apocynum*	*cannabinum*
	Asclepias	*syriaca*
Asteraceae	*Achillea*	*millefolium*
	Chrysanthemum	*leucanthemum*
	Cichorium	*intybus*
	Cirsium	*arvense*
	Crepis	*capillarus*
	Erigeron	*annuus*
	Jacobaea	*vulgaris*
	Rudbeckia	*hirta*
	Sonchus	*asper*
	Taraxacum	*officinale*
	Verbiscina	*occidentalis*
	Xanthium	unknown
Brassicaceae	*Barbarea*	*vulgaris*
	Lepidium	*virginicum*
Campanulaceae	*Triodanis*	*perfoliata*
Caryophyllaceae	*Cerastium*	*brachypetalum*
	Dianthus	*armeria*
	Myosoton	*aquaticum*
	Silene	*latifolia*
	Stellaria	*graminea*
Convolvulaceae	*Calystagia*	*sepium*
Fabaceae	*Meliotus*	*officinalus*
	Trifolium	*pratense*
	Trifolium	*repens*
	Trifolium	*campestre*
	Vicia	*sativa*
Geraniaceae	*Geranium*	*carolinianum*
Hypericaceae	*Hypericum*	*punctatum*
Iridaceae	*Sisyrinchium*	*atlanticum*
Lamiaceae	*Clinopodium*	*vulgare*
	Glechoma	*hederacea*

	Prunella	*vulgaris*
Onagraceae	*Oenothera*	*fruticosa*
Oxalidaceae	*Oxalis*	*stricta*
Polygonaceae	*Persicaria*	*longiseta*
Primulaceae	*Lysimachia*	*ciliata*
Ranunculaceae	*Anemone*	*virginiana*
	Clematis	*virginiana*
	Ranunculus	*bulbosa*
Rosaceae	*Agrimonia*	*pubescens*
	Duchesnia	*indica*
	Fragaria	*vesca*
	Geum	*canadense*
	Potentilla	*simplex*
	Potentilla	*recta*
	Rosa	*multiflora*
	Rubus	*argutus*
Rubiaceae	*Galium*	*pilosum*
	Galium	*pilosum*
	Gallium	*mollugo*
Scrophulariaceae	*Verbascum*	*thapsus*
Solanaceae	*Solanum*	*carolinense*
Umbellifers	*Daucus*	*carota*
Apiaceae	*Cryptotaenia*	*canadensis*
Verbenaceae	*Verbena*	*urticifolia*

Figure S2- Insect species documented visiting flowers at the study site. Table lists the Family, Genus, and Species of floral visitors at Hampton Creek Cove State Natural Area. Asterisks indicate morphogroups that were found not be carrying pollen and are considered non-pollinating insects.

Family	Genus/Species
Adrenidae	*Adrena sp.15*
Agromyzidae	*Agromyza sp.55*
Andrenidae	*Andrena sp. 1*
	Andrena vicina
	Perdita sp.98
Apidae	*Apis mellifera*
	Bombus sp.5
	Ceratina sp. 4
	Clisodon sp.32
	Xylocopa virginica
Asilidae	*Holcocephala sp.53*
Bombyliidae	*Villa lateralis*
	Bombylius major
Braconidae	*Eubazus sp. 31**
	Eubazus sp. 65
	Eubazus sp. 80
	Spathius elegans
Cantharidae	*Chauliognathus marginatus*
	Chauliognathus pennsylvanicus
	Podabrus sp.26
Cecidomyiidae	*Lasioptera sp.34*
Cerambycidae	*Tetraopes tetrophtalmus*
Chrysomelidae	*Altica bimarginata*
	Chrysolina quadrigemina
	Trirhabda sp.84
	Donacia sp.66
Cicadellidae	*Draeculacephala sp.89*
Colletidae	*Colletes compactus*
	Colletes sp.33
	Hylaeus modestus
Coreidae	*Euthochtha galeator*

Coreidae	*Anasa tristis*
Crabronidae	*Anacrabro ocellatus*<br>*Cerceris sp.6*<br>*Mimesa sp.99*
Curculionidae	*Hypera postica**<br>*Hypera sp.72*<br>*Odontocorynus sp.73*<br>*Sitophilus oryzae*
Formicidae	*Ant sp.*
Halictidae	*Agapostemon virescens*<br>*Augochlora pura*<br>*Lasioglossum sp.30*
Hesperiidae	*Epargyreus clarus*<br>*Euphyes dukesi*<br>*Hylephila phyleus**
Ichneumonidae	*Neorhacodes sp.79*
Leucospidae	*Leucospis affinis*
Lonchaeidae	*Dasiops sp.54*
Lygaeidae	*Lygaeus turcicus*
Megachilidae	*Megachile pugnata*<br>*Megachile sp.9*<br>*Anthidium illustre*<br>*Coelioxys octodenata*<br>*Hoplitis sp.100*<br>*Megachile gemula*
Miridae	*Collaria oculata*<br>*Halticus apterus*<br>*Lopidea sp.90*<br>*Lygus lineolaris*
Mordellidae	*Mordella atrata*<br>*Mordella marginata*
Muscidae	*Haematobia irritans*<br>*Musca domestica*
Mydidae	*Rhaphiomidas sp.94*
Myopinae	*Thecophora propinqua*
Noctuidae	*Idia sp.20*

Nymphalidae	*Clossiana bellona*<br>*Everes comyntas*<br>*Phyciodes tharos*
Oecophoridae	*Pyramidobela sp.21*
Pelecinidae	*Pelecinus polyturator*
Pentatomidae	*Cosmopepla lintneriana*
Phalacridae	*Olibrus sp.27*
Pompilidae	*Anoplius sp.42*
Reduviidae	*Phymata americana*
Rhopalidae	*Harmostes reflexulus*
Sarcophagidae	*Sarcophaga pernix*
Scarabaeidae	*Popillia japonica*
Sphecidae	*Ammophila sp. 1**<br>*Eremnophila aureonotata*
Syrphidae	*Eristalis arbustorum*<br>*Eristalis tenax*<br>*Rhingia sp.69*<br>*Sphaerophoria contigua*<br>*Syritta pipiens*<br>*Syrphus ribesii*<br>*Toxomerus marginatus*
Tachinidae	*Cylindromyia sp.47*<br>*Gymnosoma sp.49*<br>*Juriniopsis floridensis*<br>*Trichopoda pennipes*
Tephritidae	*Euaresta bella*
Tettigoniidae	*Scudderia sp.87**
Thyreocoridae	*Corimelaena obscura*
Tiphiidae	*Tiphia sp.*
Vespidae	*Euodyerus hidalgo*<br>*Polistes dominula*<br>*Vespula malculifrons*

Figure S3- Phenologies of plant species at the study site throughout the sampling period (13 weeks). Each line represents flowering time for a different plant species between May 15 and August 13.

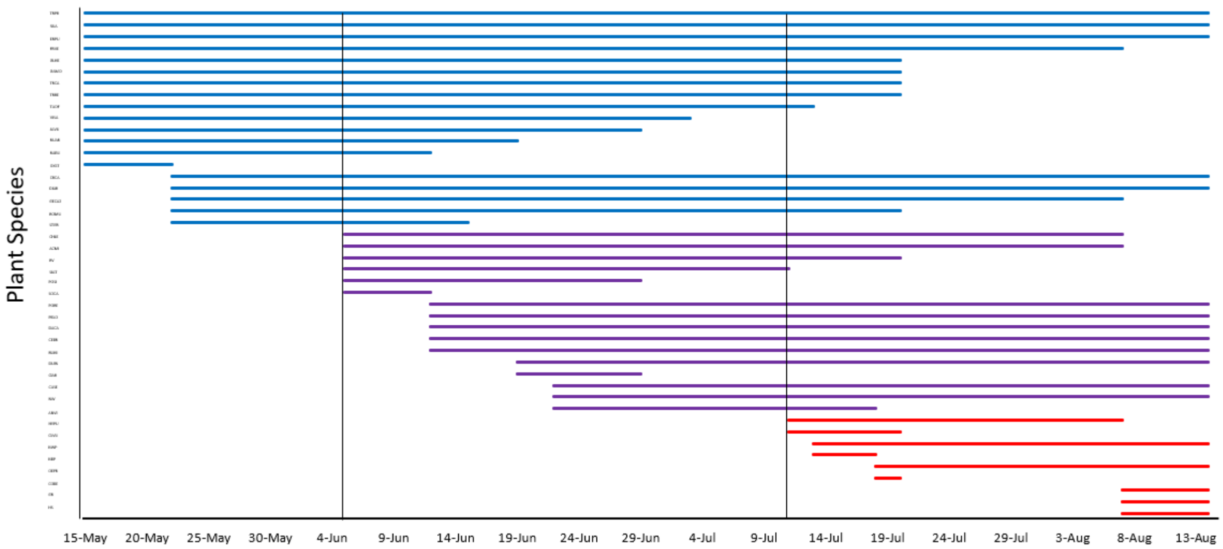

Figure S4- Rarefaction curves representing sampling completeness for each network type a) floral visitation-whole, b) pollen transport-whole and for each time period within a season, c) early season (ES), d) middle season (MS) and e) late season (LS). Sampling effort (days) is represented in the 'x axis' and number of uinique plant-pollinator interactions are represented on the 'y axis'. Solid lines represent the onsrved number of interactions and dotted lines represent the Jackknife and bootstrap interaction richness estimators (see methods). In general we sampled between 64-80% of all interactions across network types and time periods.

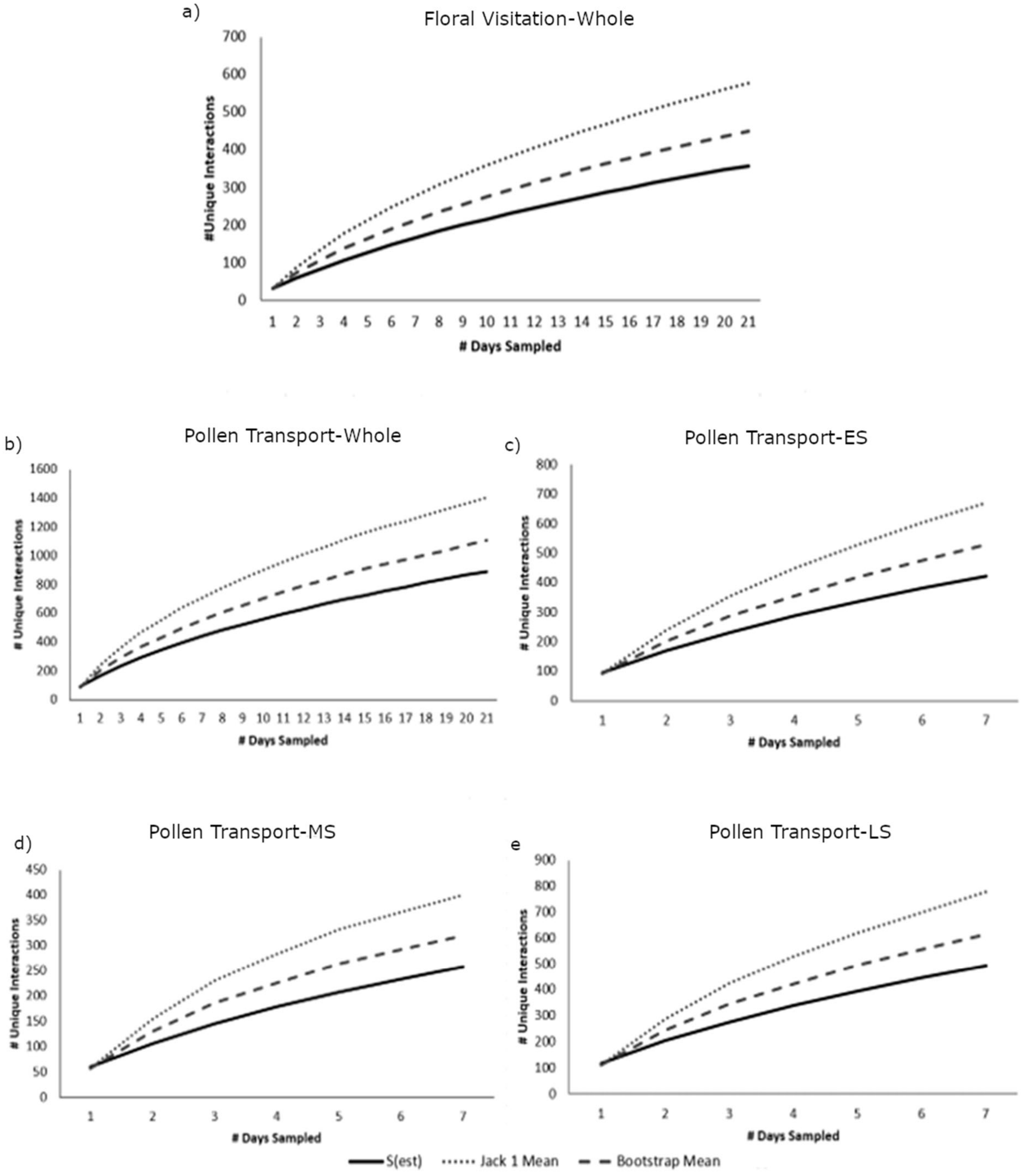

Figure S5- Bipartite network of ‘non-pollinating’ interactions between plants and insects. The network was generated by subtracting ‘pollen-transport’ interactions from the ‘floral visitation’ network. The reaiming interactions are those that represent insects that visit flowers but do not participate in pollen transport but may be biologically relevant in other ways (e.g. microbial transport, nectar robbers). . Insect morphogroups are represented on the top and plants at the bottom. Interactions are represented by the lines between nodes. The thickness of the lines reflect the frequency of those interactions

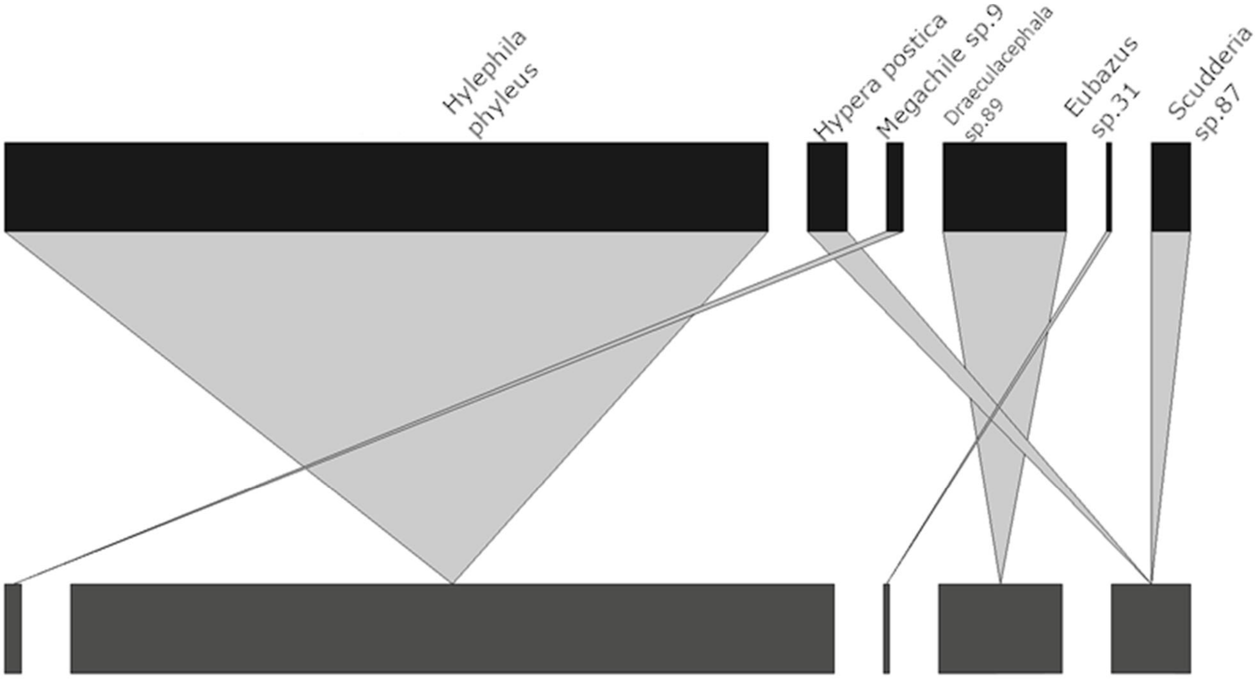

Supplement: plab062_suppl_Supplementary_Materials [file plab062_suppl_supplementary_materials.pdf]
